# Lymphoma in Miniature Dachshunds: A retrospective multicenter study of 108 cases (2006‐2018) in Japan

**DOI:** 10.1111/jvim.16455

**Published:** 2022-05-27

**Authors:** Kenji Rimpo, Miyuki Hirabayashi, Aki Tanaka

**Affiliations:** ^1^ Saitama Animal Medical Center Saitama Japan; ^2^ Nippon Veterinary Life Science University Tokyo Japan

**Keywords:** gastrointestinal lymphoma, lymphoma, miniature dachshunds, Mott cell

## Abstract

**Background:**

Miniature Dachshunds (MD) are predisposed to lymphoma with disease onset of young age and long‐term survival.

**Objectives:**

To compare clinical features and survival time of lymphoma in MD and non‐MD.

**Animals:**

One hundred and eight MDs with lymphoma and 149 non‐MD breed dogs with lymphoma were included in the study.

**Methods:**

This was a retrospective multicenter observational study. Lymphoma was classified based on signalment, histopathology/cytology, and anatomical site of the disease. For each type of lymphoma, median survival time was analyzed by Kaplan‐Meier estimates and life table analysis. Prognostic factors for large‐cell gastrointestinal lymphoma (LGIL) were analyzed using Cox regression.

**Results:**

Gastrointestinal lymphomas were more common in MDs (53/108) compared to non‐MDs (41/149). The multicentric lymphoma was most common in non‐MD breed dogs (74/149) compared to MDs (33/108). The median age that dog developed lymphoma in MD and non‐MD were both 10 years old; however, lymphomas were more frequently observed in younger dogs (<4 years) in MDs (20/108) compared to non‐MDs (9/149; *P* = .002). Seventy percent were diagnosed with B‐cell with median age of diagnosis was 3 (1‐14) years. Mott cell differentiation was observed in 6 dogs. Age <4 years and B‐cell phenotype were significant factors for longer survival time in MD with LGIL.

**Conclusions and Clinical Importance:**

Lymphomas in MDs involved gastrointestinal lesions at higher frequency compared to other dog breeds examined. B‐cell lymphoma was more common in early‐onset LGIL in MD and cases that involved Mott cell differentiation were observed. Awareness of this specific presentation of lymphoma in dogs will possibly affect the treatment decision process for the owners of MD with LGIL.

**Abbreviations**
CD: cluster of differentiation
CI: confidence interval
CL: cutaneous lymphoma
COP: cyclophosphamide, vincristine, prednisolone
GIL: gastrointestinal lymphomas
LML: large cell multicentric lymphoma
L‐CHOP: L‐asparaginase, cyclophosphamide, doxorubicin, vincristine, prednisolone
LLP: L‐asparaginase, lomustine, prednisolone
MST: median survival time
MD: miniature dachshund
ML: multicentric lymphomas
PARR: antigen receptor rearrangements
SD: standard deviation
ST: survival time
VCS: Veterinary Cancer Society

## 1 INTRODUCTION

Multicentric lymphomas (ML) account for approximately 80% of cases of lymphoma in dogs; however, gastrointestinal lymphomas (GIL) account for 5% to 7%.^1^, ^2^, ^3^, ^4^ The median survival time (MST) for ML and GIL is 10 to 12 months and 0.5 to 2.5 months, respectively.^5^, ^6^, ^7^, ^8^, ^9^ The immunophenotype of tumor cells is 1 of the reasons for the short survival time of dogs with GIL.^17^, ^18^ Among ML, B‐cell lymphoma account for 70% to 80% and have a MST of 11 to 13 months, while T‐cell lymphoma is chemoresistant and had a MST of 5 to 5.3 months.^5^, ^10^, ^11^, ^12^ T‐cell lymphoma account for 63% to 91% of GIL in dogs^6^, ^7^, ^8^, ^13^, ^14^; the higher proportion of T‐cell lymphoma might be 1 factor associated with shorter MST. Moreover, there have been a small number of cases of B‐cell GIL with long‐term survival.^15^, ^16^ Furthermore, colorectal lymphomas in dogs often originate in B‐cells and survive long term.^17^, ^18^ A case report found that a miniature dachshunds (MD) dog had a good prognosis and different characteristics than usual and occur at a younger age (mean age = 4 years).^19^ However, their study did not assess the immunological characteristics nor mentioned Mott cells. It is clinically relevant to demonstrate the difference, if any, of response to the treatment and survival time between lymphoma in MD and other breed dogs to better inform clients in MD diagnosed with lymphoma. This study therefore aimed to compare the clinical characteristics of lymphoma in MD with those of other dog breeds.

## 2 MATERIALS AND METHODS

### 2.1 Study design

This was a retrospective multicenter observational study performed at 11 animal hospitals in Japan.

### 2.2 Animals

MD dogs diagnosed with lymphoma at a referral veterinary hospital and 10 other veterinary clinics and hospitals in Japan between 2006 and 2018, and non‐MD dogs of other breeds diagnosed with lymphoma during the same period at the referral veterinary hospital were included in this study. Dogs that did not undergo chemotherapy or for which survival time (ST) was unknown were excluded from the survival analyses.

### 2.3 Review of medical records

The age, sex, weight, anatomical site of the disease, malignancy by histopathology or cytology, immunological classification, complete remission rate, first remission, and survival time were assessed based on medical records. Lymphoma was classified as per cell morphology and size using a clinical or diagnostic pathologist. Small cell lymphoma was histopathologically diagnosed using surgical excision biopsy or endoscopic biopsy specimens. Immunological classification was performed by immunohistochemistry using CD3 or CD79 antigens or by polymerase chain reaction (PCR) for antigen receptor rearrangements (PARR). Complete remission was determined by the attending veterinarian based on physical examination results, chest and abdominal X‐ray, abdominal ultrasound, and CBC results. Complete remission for GI lymphoma was determined by abdominal ultrasound when there was no lesion detected. Complete remission for multicentral lymphoma was determined by evaluating superficial lymph nodes through palpation, evaluating thoracic lymph nodes and lungs through thoracic radiograph, and evaluating abdominal lymph nodes, liver and spleen through abdominal ultrasound. As for cutaneous lymphoma, skin lesion was evaluated additional to the above examinations. First remission time was defined as the time from the day of complete remission until the confirmation of relapse. Regardless of the cause of death, survival time was defined as the period from date of diagnosis to death or euthanasia.

### 2.4 Statistical analysis

Using the Shapiro‐Wilk test, continuous data were assessed for the normality of distribution. Normally distributed continuous variables were reported as mean ± SD, and non‐normally distributed continuous variables were reported as median (range). Using the Kruskal‐Wallis test, age of occurrence of lymphoma was compared. The chi‐square test was used to analyze sex difference of study dogs and number of dogs for each lymphoma types. The Kolmogorov‐Smirnov test was used to analyze the distribution of age between MD and non‐MD groups. Proportion test was performed to compare complete remission proportion between groups. The complete remission and first remission rates were compared using the Kruskal‐Wallis test. The Kaplan‐Meier analysis and log‐rank test were performed to analyze the survival period and first remission time among groups. Median survival period and 95%CI were computed by survival analysis and those that did not reach 50% survival probability at the end of the study, life table analysis was performed to analyze median survival period at a survival probability of 50% with 95%CI. Dogs that were still alive at the end of study, lost to follow‐up, or died of other reason than lymphoma was censored. Univariate Cox regression analyses were performed to evaluate the prognostic factors for large cell gastrointestinal lymphoma in MD. Those parameters with a *P* < .10 on univariate analysis were put into the multivariate analysis to construct a final model by backward elimination. Stata Statistical Software: Release 16 (StataCorp, College Station, Texas) was used in all analyses. For statistical estimation and inferences, 2‐sided hypotheses and tests were used with a 5% significance level.

### 2.5 Ethical statement

This was a retrospective observational study using hospital data at animal hospitals. Hence, informed consent from the animal owners was not applicable here. The study was approved by hospital boards at Saitama Animal Medical Center (Approval number: 20191001, approval date: November 15, 2019). All dogs were treated and evaluated by board‐certified specialists at the hospital and provided with customary high standards of medical care and welfare.

## 3 RESULTS

A total of 257 dogs were included in this study. One hundred eight dogs were MDs and 149 dogs were non‐MDs. The breed representation for non‐MDs diagnosed with lymphoma is described in Table 1. The median age (range) for MDs and non‐MDs were 10 (1‐18) and 10 (1‐17) years old, respectively. Twenty out of 108 dogs and 9 out of 149 dogs were <4 years in MDs and non‐MDs, respectively (*P* = .02). The median body weight (range) for MD and non‐MD were 6 (3‐11) kg and 11(2‐48) kg, respectively (*P* = .0001). Sixty dogs were male and 47 were female in MD, and 69 were male and 57 were female in non‐MD (*P* = .792). No dog was lost to follow up in this study.

**TABLE 1:** The breed representation for non‐MD group (n = 149) diagnosed with lymphoma during 2006 to 2018

Dog breed	Number of dogs	Percentage
Afghan hound	1	0.7
American cocker spanial	2	1.3
Belgian shepherd dog	1	0.7
Borzoi	1	0.7
Bull terrier	1	0.7
Cairn terrier	1	0.7
English bulldog	1	0.7
English cocker spaniel	3	2.0
French bulldog	4	2.7
German shepherd	1	0.7
Italian gray hound	2	1.3
Jack Russell terrier	1	0.7
Kai‐ken	3	2.0
Labrador retriever	9	6.0
Maltese	2	1.3
Miniature pinscher	3	2.0
Miniature schnauzer	2	1.3
Norfolk terrier	1	0.7
Pug	3	2.0
Shih Tzu	2	1.3
West highland terrier	1	0.7
Yorkshire terrier	6	4.0
Barneys mountain dog	3	2.0
Beagle	4	2.7
Border coli	3	2.0
Chihuahua	11	7.3
Golden retriever	17	11.3
Mix	17	11.3
Papillon	1	0.7
Shetland sheepdog	6	4.0
Shibainu	14	9.3
Toy poodle	3	2.0
Welsh corgi	19	12.7

Small cell gastrointestinal lymphoma was observed in 13 dogs (10 MDs and 3 non‐MDs), and small cell multicentric lymphoma was observed in 2 non‐MDs. There was no small cell cutaneous lymphoma recognized in this study. Among 108 MD diagnosed with lymphoma, GIL was most common (n = 52), followed by multicentric (MCL; n = 33), cutaneous (n = 11), hepatosplenic (n = 6), anterior mediastinal (n = 1), and other types of lymphoma (n = 5). Among the 149 non‐MDs diagnosed with lymphoma, MCL was most common (n = 74), followed by GIL (n = 41), cutaneous (n = 14), hepatosplenic (n = 11), mediastinal (n = 3), and other types of lymphomas (n = 6; Table 2). The percentage of GIL was significantly higher in lymphoma in MD than in non‐MD s (*P* = .0178). There was no significant difference between other anatomic sites for the MD and non‐MD groups. The difference in the presentation between MD and non‐MD and substage were not determined in this study.

**TABLE 2:** Anatomical site of lymphoma observed in this study dogs (MD = 109 and non‐MDs = 149)

	Multicentric	Gastrointestinal	Cutaneous	Liver/spleen	Mediastinum	Other
MD (n = 109)	33	52	11	6	1	5
non‐MDs (n = 149)	74	41	14	11	3	6

### 3.1 Investigation of gastrointestinal lymphoma in MD

Among the 52 MD diagnosed with gastrointestinal lymphoma (GIL), 42 were large cell gastrointestinal lymphoma (LGIL) and 10 were small cell gastrointestinal lymphoma (SGIL). Among the non‐MDs with GIL, 38 were LGIL and 3 were SGIL. Because there were only a few cases of SGIL, survival time was compared in dogs with only LGIL.

### 3.2 Comparison of age of LGIL onset

**FIGURE 1:**
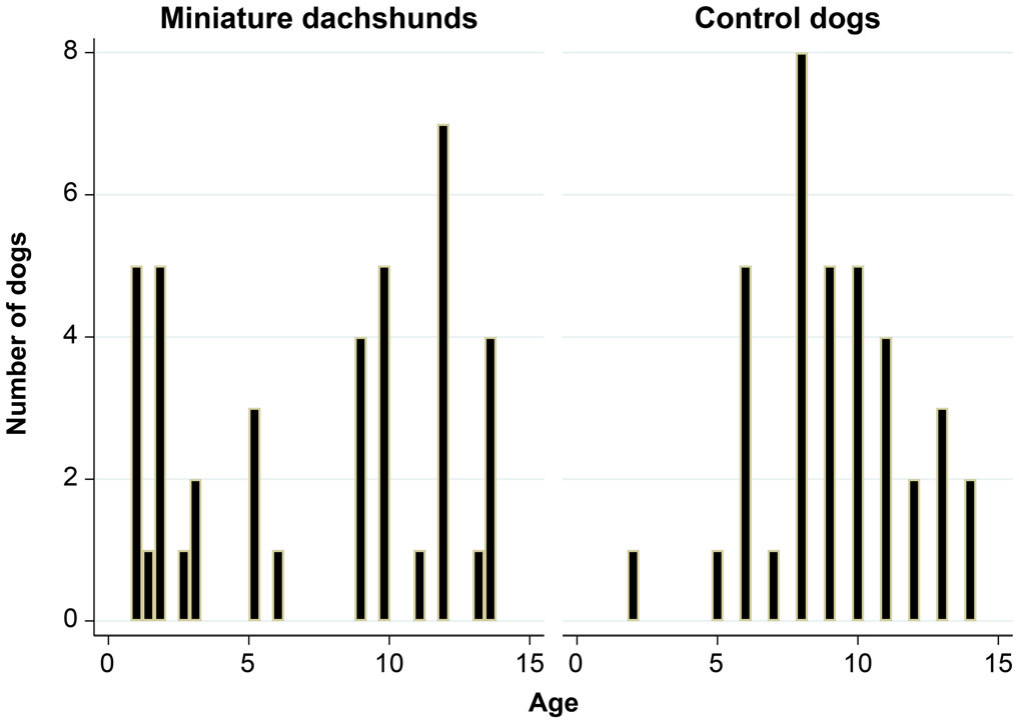
Histogram of distribution of age at onset of LGIL in MD and non‐MDs. There were more cases of early onset in MD than the non‐MD dogs, resulting in bimodal distribution (*P* = .01)

No significant (*P* = .329) difference in median age of LGIL onset was seen in MD dogs (median 9 years, range 1‐14) and non‐MD dogs (median 9 years, range 2‐14; *P* = .3239). However, the comparison of age distributions resulted in higher incidence of early‐onset disease, and a bimodal distribution of age at onset in MD compared to the incidence age distribution in non‐MDs (*P* = .01; Figure 1).

### 3.3 Immunological classifications of LGIL

Of 42 MD with LGIL, immunological classifications were performed in 28 cases. Of the 28 cases, 20 (71%) were diagnosed with B‐cell and 8 (29%) were diagnosed with T‐cell LGIL. The median age at the onset of 8 cases diagnosed with T‐cell type was 12 years (range: 9‐15 years), whereas the median age of 20 cases diagnosed with B‐cell type was 3 years (range: 1‐14 years; *P* = .0005). Mott cell differentiation of neoplastic cells was observed in 6 of 20 cases (Table 3). The median age of these 6 dogs was 2 years (range: 1‐5 years). Immunological classifications were assessed in only 27 dogs of 38 non‐MD LGIL and resulted in 18 (67%) of T‐cell and 4 dogs with B‐cell lymphoma (15%). Mott cell differentiation was observed in 1 of the 4 dogs, which was a 2‐year‐old female MD mix. As such, LGIL in MD represented a significantly higher incidence of B‐cell LGIL than the non‐MDs (*P* = .001).

**TABLE 3:** Immunological classification of large cell gastrointestinal tract lymphoma in study dogs (MD = 29, non‐MDs = 27)

	T‐cell	B‐cell (Mott cell)	Non‐T/Non‐B
MD (n = 29)	9	20(6)	0
non‐MDs (n = 27)	18	4	5

*Note*: The number of B‐cell lymphoma was significantly higher in MD than in non‐MDs (*P* = .001).

### 3.4 LGIL onset site

The LGIL site in MD was observed in the small bowel (n = 28), ileocecal junction (n = 5), duodenum (n = 1), and stomach (n = 1); 1 dog was reported with a mass in 3 sites, the small bowel, large bowel and tonsils, and 6 MD with LGIL were observed to have the swelling of mesenteric nodes only and without distinct mass formation in the gastrointestinal tract. In LGIL in non‐MDs, mass formation was observed in the small bowel (n = 24), duodenum (n = 5), stomach (n = 1), stomach and duodenum (n = 1), duodenum and ileum (n = 1), and cecum (n = 1), while only swelling of the mesenteric lymph nodes was observed in 5 dogs. No dogs exhibited lesions in the large bowel. No significant difference in onset localization/site was seen between MD and non‐MD dogs with LGIL (*P* = .1).

### 3.5 Treatment for LGIL

Chemotherapy was administered to 34 of 42 MD cases, of which surgery was performed before chemotherapy in 6 cases. The chemotherapy protocol was selected by the attending veterinarian. Twenty‐four dogs were administered L‐CHOP (L‐asparaginase, cyclophosphamide, doxorubicin, vincristine, and prednisolone) for initial treatment, 4 with COP, and 3 with LLP (L‐asparaginase, lomustine, prednisolone), and 2 with vincristine and prednisolone. Thirty‐four of the 38 non‐MD cases underwent chemotherapy, and 4 underwent surgical resection before chemotherapy. Chemotherapy comprised L‐CHOP for 22 dogs, COP for 3 dogs, LPP for 6 dogs, chlorambucil and prednisolone for 2 cases, and prednisolone and melphalan for 1 case.

### 3.6 Complete remission rate and first remission time of LGIL

The complete remission rates for MD and non‐MDs were 68% (23/34) and 29% (10/34), respectively, resulting in a statistically significant difference (*P* = .002). MD was divided into cases that lived <4 years and ≥4 years, which resulted in 93% (13/14) and 50% (10/20), respectively, thus indicating a significant difference (*P* < .0001). The median first remission time of MD (n = 19) and non‐MDs (n = 10) that achieved complete remission were 672 (13‐5900) and 88 (14‐126) days, respectively, thus indicating a significant difference (*P* = .0009). There was a significance difference (*P* = .0043) between median first remission time of MD <4 years (n = 13; median 1223 days, range 13‐5900) and MD ≥4 years (n = 10; median 270 days, range 24‐790).

### 3.7 LGIL survival time

At the end of the study period, 13 of 34 LGIL cases that underwent chemotherapy were still surviving. Of 21 MDs that died, the cause of death was believed to be lymphoma in 17 MDs, cause other than lymphoma in 2 cases, and cause of death was unknown in 2 cases. The survival time was significantly longer (*P* = .0001) for MD with LGIL with the MST1503 days (survival probability of 50% with 95%CI: 30‐57) compared to non‐MDs with the MST 56.7 days (survival probability of 50% with 95%CI: 32‐65; Figure 2).

**FIGURE 2:**
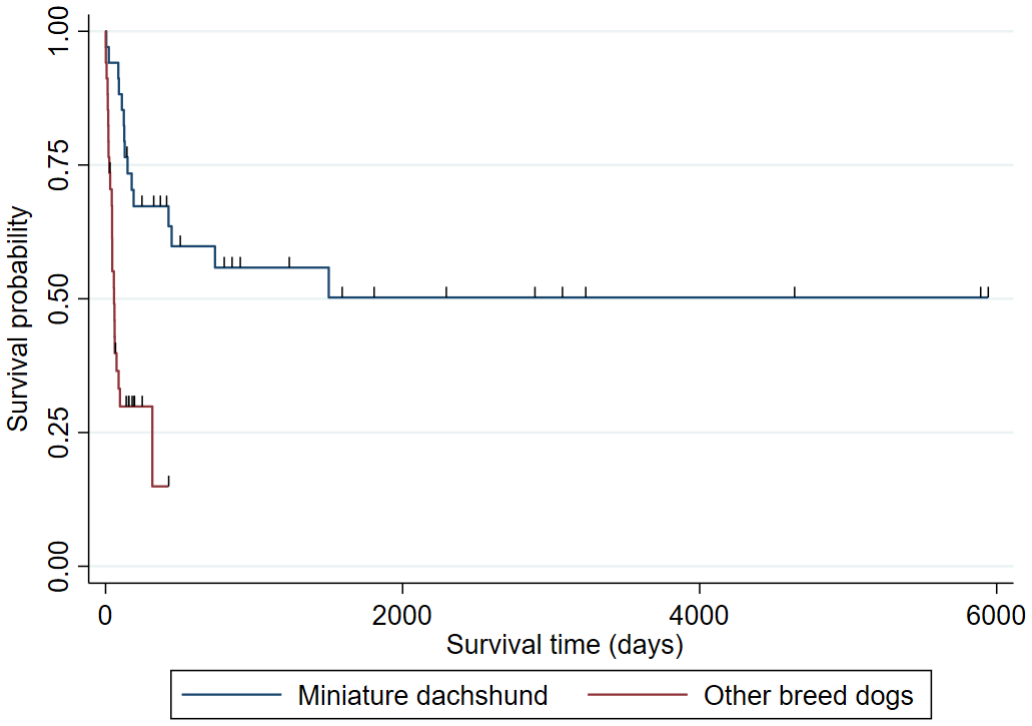
Kaplan‐Meier curve of overall survival time of LGIL in MD and the non‐MD dogs. The MST of MD was 1503 days (survival probability of 50% with 95%CI: 30‐57). The MST of non‐MD dogs, moreover, was 56.7 days (survival probability of 50% with 95%CI: 32‐65), showing that the survival time of LGIL was significantly longer in MD (*P* = .0001)

### 3.8 Influence of age on LGIL

**FIGURE 3:**
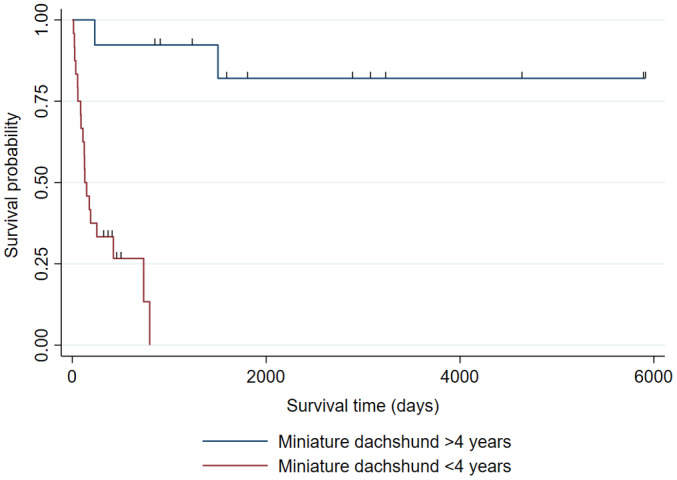
Kaplan‐Meier curve of LGIL in MD by survival time ≥4 years versus <4 years. Ten of the MD with onset at <4 years (n = 14) were surviving at the end of the study period; however, their survival time did not reach MST. MST of MD with onset at ≥4 years (n = 20) was 148 days (95%CI: 90‐424), showing that the MD with onset at <4 years had significantly longer survival time than the MD with onset at ≥4 years (*P* < .0001)

Ten out of 14 of the MDs with onset at <4 years were surviving at the end of the study period. Among these 14 cases, the immunophenotype was assessed in 10 cases; all were B cell, while it was unknown in 4 cases because they were not tested. The MST of MDs with onset at ≥4 years (n = 20) was 148 days (95%CI: 90‐424). Among the 15 of these 20 cases that were assessed for immunophenotype, 9 were B‐cell and 6 were T‐cell. All 6 cases with T‐cells were ≥9 years. The immunophenotype was unknown in 5 cases that were untested. The survival time was significantly longer in MDs diagnosed at <4 years than those diagnosed at ≥4 years (*P* < .001; Figure 3).

**FIGURE 4:**
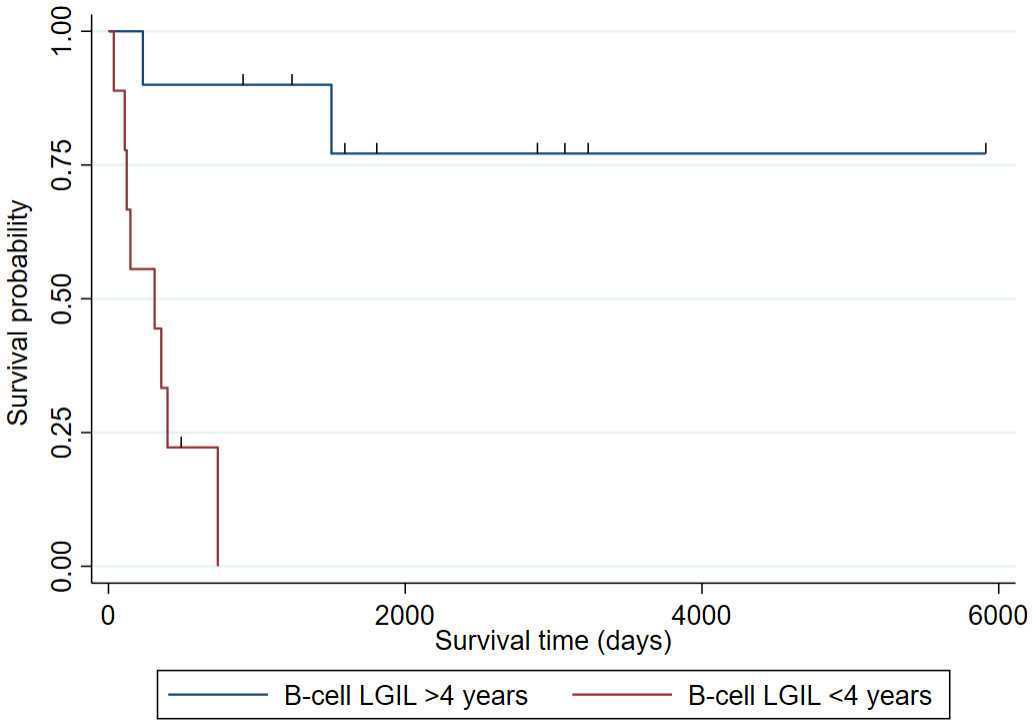
Kaplan‐Meier curve of the survival time of MD with B‐cell LGIL onset at ≥4 years versus <4 years. The survival time of MD with B‐cell LGIL diagnosed at <4 years did not reach the median. However, among dogs diagnosed at ≥4 years, the MST was 229 days (survival probability of 50% with 95%CI: 17‐76), showing that the survival time of MD diagnosed at <4 years was significantly longer (*P* = .0011)

Additional comparisons were made in the survival time of B‐cell LGIL as per age by MDs <4 years (n = 10) and ≥4 years at onset (n = 9). The survival time of MD <4 years with B‐cell LGIL did not reach MST. However, the MST of MD ≥4 years was 229 days (survival probability of 50% with 95%CI: 17‐76); the survival time of MD <4 years was significant longer (*P* = .0011; Figure 4).

### 3.9 Comparison of survival time of LGIL in MD according to immunophenotype

The survival of LGIL in MD was assessed as per immunophenotype. The survival time was significantly longer (*P* = .0171) for MD with B‐cell LGIL (n = 19) with the MST 1313 days (survival probability of 50% with 95%CI: 23‐67) compared to T‐cell LGIL (n = 6) with the MST 144 days (survival probability of 50% with 95%CI: 11‐80; Figure 5).

**FIGURE 5:**
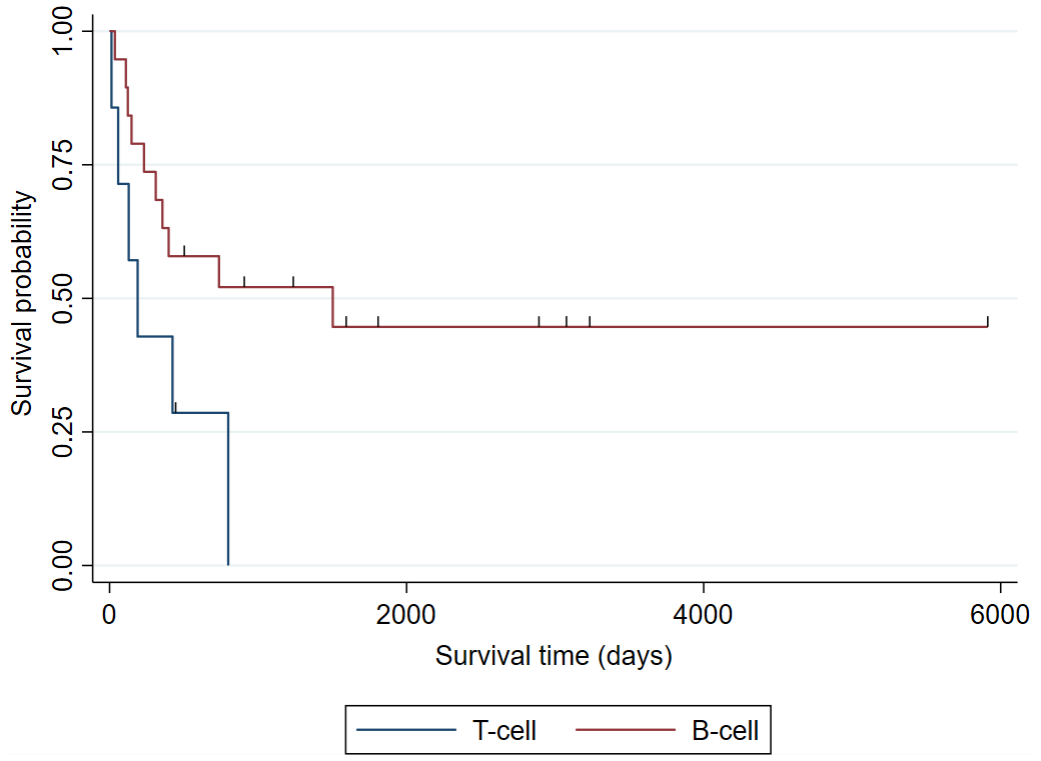
Kaplan‐Meier curve of the survival time of MD with LGIL as per B‐cell versus T‐cell phenotype. Median survival time of MD with B‐cell LGIL (n = 19) and T‐cell LGIL (n = 6) were 1313 days (survival probability of 50% with 95%CI: 23‐67) and 144 days (survival probability of 50% with 95%CI: 11‐80), respectively, showing that the survival time for B‐cell LGIL was significantly longer (*P* = .0171)

### 3.10 Comparison of survival time according to Mott cell differentiation of tumor cells

Of the 19 B cell LGIL, the MST of 6 dogs with Mott cell differentiation was 1354 days (survival probability of 50% with 95%CI: 10‐81); however, it was 377 days for the 13 MD without Mott cell differentiation (at survival probability of 50% with 95%CI: 22‐73); the difference was not significant (*P* = .3805).

### 3.11 Analysis of the prognosticators of LGIL in MD

The univariate Cox regression of prognosticators of LGIL in MD resulted in age <4 years (*P* < .0001) and B‐cell phenotype (*P* = .025) as significant factors (Table 3).

### 3.12 Results of nongastrointestinal lymphomas

All 33 of MDs with multicentric lymphoma (ML) were large cell multicentric lymphoma (LML), and their median age at onset was 11 (2‐18) years. The median age at onset of LML in the 76 non‐MD dogs was 9 (7‐17) years. Twenty‐two out of 33 MDs and 46 out of 56 non‐MDs died in this study. There was no significant difference in MST (*P* = .1457) nor in the age distribution (*P* = .319) between the 2 groups. (Two of the non‐MDs comprised small cell ML and were therefore excluded from the analysis.)

Immunophenotype was assessed in 25 of 33 MD with LML, of which 72% (n = 18) were B‐cell and 20% (n = 5) were T‐cell, and clonality was not detected in 2 cases. Immunophenotype was assessed in 53 of 74 non‐MDs, of which 72% (n = 38) were B‐cell and 26% (n = 14) were T‐cell, while clonality was not detected in 1 case. There was no significant difference between these 2 groups (*P* = .923) in immunophenotype (Table 4).

**TABLE 4:** The univariate Cox regression analysis of the association between time to death and age, sex, weight, cell type, and Mott cell in miniature dachshunds (MD) with LGIL

	Time to death		95%CI	*P*‐value
	Number of dogs	Incident rate ratio		
Age				
Less than 4 years old (<4 years)	14	Reference		
Above 4 years old (>4 years)	20	39.90	5.02‐316.87	<.0001
Sex				
Male	18	Reference		
Female	17	1.2	0.52‐2.70	.687
Weight	37	1.1	0.80‐1.43	.629
Cell type				
T‐cell	6	Reference		
B‐cell	19	0.29	0.10‐0.85	.025
Mott cell				
No Mott cell	13	Reference		
With Mott cell	6	0.55	0.14‐2.19	.400

Twenty of the 33 MDs with LML underwent L‐CHOP or CHOP therapy, of which 5 underwent COP and 2 underwent doxorubicin monotherapy. Six were treated by symptomatic therapy, including prednisolone. Forty‐five of the 74 non‐MD cases were treated by L‐CHOP or CHOP, of which 7 underwent COP and 2 underwent doxorubicin monotherapy. Twenty cases were treated with symptomatic therapy only.

The complete remission rates of MDs and non‐MDs with LML that underwent chemotherapy were 82% (24/29) and 75% (42/56), respectively, resulting in no significant difference between MDs and other breeds (*P* = .291). The first remission time of MDs and non‐MDs were 170 (range: 14‐1674) and 208 (range: 20‐984) days (*P* = .4769). MST for MD was 201 days (95%CI: 120‐402) and 178 days for non‐MDs (95%CI: 108‐402; *P* = .9157).

The median age at cutaneous lymphoma (CL) onset was 11.5 years for MD (range: 7‐15, n = 11) and 10 years for non‐MDs (range: 6‐14, n = 14; *P* = .4259). There was no significant difference in age distribution (*P* = .955).

The immunophenotype was assessed in 9 of the 11MD with CL, out of which 78% (n = 7) comprised T‐cell and 11% (n = 1) of B‐cell, and clonality was not detected in 1 case. The immunophenotype was tested in 11 of 14 non‐MDs, of which 73% (n = 8) were T‐cell and 18% (n = 2) were B‐cell, and clonality was not detected in case 1. No significant difference was reported between these 2 groups (*P* = .679).

Of the 11 MD with CL, 10 were treated by chemotherapy. Lomustine and prednisolone induction therapy were administered in 7 cases, chorambucil and prednisolone in 2 cases, and COP therapy was administered in 1 case. Thirteen of the 14 non‐MDs with CL were administered chemotherapy, out of which 9 were administered lomustine and prednisolone, 3 were administered chorambucil and prednisolone, and 1 was treated by CHOP.

The complete remission rate of dogs with CL that underwent chemotherapy was 20% (2/10) for MDs and 30% (4/13) for non‐MDs (*P* = .066). All the MDs and 9 out of 14 non‐MDs died in this study. The MST was 60 days for MDs (survival probability of 50% with 95%CI: 20‐74) and 102 days for non‐MDs (95%CI: 23‐608; *P* = .7586).

## 4 DISCUSSION

This study demonstrated that the percentage of gastrointestinal lymphoma was higher in MDs than in other dog breeds. The reasons for this remain largely unknown; however, the genetic factors of MDs could be playing a role. One observation that supported this was the difference in age of onset of LGIL in MDs. Although there was no significant difference in the median age of onset in MDs and non‐MDs, the age of onset in non‐MDs was unimodal whereas the distribution was bimodal in MD, with a peak at a relatively young age of <4 years, and a second peak at >10 years. It is possible that the genetic background specific to MD is involved for the population with early onset. According to the 2006 VCS oral presentation by Setoguchi et al. (unpublished article), the mean age of 38 MD with lymphoma was 4 years. The putative reason for this low mean age of MDs with lymphoma is the large increase in the total population of young MDs because the dog breed gained widespread popularity in Japan at the time. LGIL in dogs in general were characterized by a low response rate (56%) and short survival time of 0.5 to 2.5 months.^5^, ^6^, ^7^, ^8^, ^9^ The MST of non‐MDs was (56 days) consistent with previous reports on LGIL in dogs in general. The cut‐off of 4 years was used in this study for MD diagnosed with LGIL as per the study by Setoguchi et al., as well as compared the survival time of MD diagnosed with LGIL at <4 years and ≥4 years. This analysis resulted in a significantly longer survival time of MD with LGIL onset at <4 years; however, it was possible that their longer survival was associated with other factors related to young age such as lower rates of comorbidities involving diseases in other organs and better tolerance of chemotherapy. Among the 14 MDs with LGIL diagnosed at <4 years, 10 dogs that were assessed for immunophenotype were B‐cell types. Moreover, 6 diagnosed with T‐cell LGIL were aged 10 years or older. These results suggested that a higher proportion of GIL in MD with early‐age onset was B‐cell and a higher proportion of late onset lymphoma was T‐cell type. The higher rates of B‐cell immunophenotype in young MD with LGIL might be associated with their longer survival. T cell phenotype accounted for the majority (63%‐91%) of LGIL in dogs reported to date.^6^, ^7^, ^8^, ^13^, ^14^ T‐cell phenotype accounted for 67% of LGIL in non‐MD dogs in this study, consistent to previous reports. The converse was true in MDs; however, with 69% confirmed to be B‐cell phenotype. In a retrospective study of 11 dogs with rectal lymphoma, the mean survival time was 1697 days, which was shorter than the MST. Survival time was significantly longer in dogs that underwent chemotherapy. An immunohistochemistry test was performed in 10 of these 11 dogs, revealing that all were B cell phenotypes.^17^ In another retrospective study of lymphoma associated with colon in dogs, immunophenotype was assessed in 26 of 31 cases, of which 24 were B‐cell lymphoma, and their MST was 61.5 months.^18^ Because there were only 2 cases of non‐B‐cell lymphoma in that study, it was not detected as a prognostic factor. However, there was a high likelihood that this difference in immunophenotype was associated with the long‐term survival of lymphoma in the colorectum. No cases of colonic lymphoma were observed in the non‐MD dogs in this study. The anatomic site of LGIL in MD was the small bowel and mesenteric nodes in the majority; as such, there were no MD with lesions localized in the colon. Therefore, it did not seem that the high rate of B‐cell lymphomas in MDs could be attributed to the high rate of LGIL of colon in MDs. Mott cell differentiation was observed in the tumor cells of 6 of 19 B‐cell LGIL in MDs. Mott cells are plasma cells packed with Russel bodies (saccules containing immunoglobulins) in the cytoplasm that were first described by Russell in 1890.^20^ Initially, the origins or significance of these cells were unknown; however, Mott hypothesized in 1901 that the cells that were identified in the brains of monkeys infected by tripanosomes were plasma cells, reflecting a state of chronic inflammation.^21^ Today, Mott cells are believed to appear in response to immunoglobulin overproduction or hyposecretion and have been observed in various inflammatory and neoplastic diseases. One such example was lymphoma with Mott cell differentiation, which was characterized by the abundance of Mott cells. In dogs, Kodama et al. reported a case of a 1‐year‐old MD in 2008.^19^ The incidence of Mott cell differentiation is rare and have only been reported in 6 dogs and 1 ferret in recent studies.^22^, ^23^, ^24^, ^25^, ^26^ Although details in other breeds are unknown because the reports are limited, a comparison with the outcomes of this study seemed to suggest that GIL presenting Mott cell differentiation in MD might show different behaviors than in other dog breeds. Our comparison of survival time as per the presence or absence of Mott differentiation revealed in no significant difference. However, this outcome could originate from a type II error, thus additional investigation of a larger sample is warranted. There was no significant difference between the non‐MDs and MDs in the age at onset, immunophenotype, responsiveness to chemotherapy, or survival time of MIL and LC. Recently, reports had demonstrated the geographic differences in the occurrence of lymphoma in dogs among countries. To our knowledge, miniature dachshunds were not found to be predisposing breed in the reports from the Western countries and there is no report describing characteristics of lymphoma in miniature dachshunds. Our report might have characterized miniature dachshunds specific to Japan.^27^, ^28^

The limitations of this study were inherent to its retrospective design and include small sample size, variable staging and treatment regimens, as well as lack of standardized follow‐up. While the population of MD was collected from multiple centers, the non‐MD dogs were exclusively selected from the referral veterinary hospital, which could have resulted in sampling bias. In addition, any consequences of surgery was not examined in this study that were performed before chemotherapy.

As per the results of analyses performed in this study, lymphomas in MDs involved gastrointestinal lesions compared to other dog breeds. The onset of LGIL in MD was bimodally distributed because onset had occurred at a young age in many dogs. Finally, B‐cell lymphoma was more common in early‐onset LGIL in MD and cases that involved Mott cell differentiation were observed. The veterinarian awareness of this specific presentation of lymphoma in dogs will possibly affect the treatment decision process for the owners of MD with LGIL. Aggressive chemotherapy for LGIL in MD might be effective for long‐term survival.

## CONFLICT OF INTEREST DECLARATION

Authors declare no conflict of interest.

## OFF‐LABEL ANTIMICROBIAL DECLARATION

Authors declare no off‐label use of antimicrobials.

## INSTITUTIONAL ANIMAL CARE AND USE COMMITTEE (IACUC) OR OTHER APPROVAL DECLARATION

Approved by hospital boards at Saitama Animal Medical Center (approval number: 20191001, approval date: November 15, 2019).

## HUMAN ETHICS APPROVAL DECLARATION

Authors declare human ethics approval was not needed for this study.

## References

[1] Jacobs R , Messick J , Valli V . Tumors of the hemolymphatic system. Tumors in Domestic Animals. Blackwell publisher, USA, Vol 4; 2002:119‐198.

[2] Vezzali E , Parodi A , Marcato P , et al. Histopathologic classification of 171 cases of canine and feline non‐Hodgkin lymphoma according to the WHO. Vet Comp Oncol. 2010;8:38‐49. 20230580 10.1111/j.1476-5829.2009.00201.x

[3] Ponce F , Marchal T , Magnol J , et al. A morphological study of 608 cases of canine malignant lymphoma in France with a focus on comparative similarities between canine and human lymphoma morphology. Vet Pathol. 2010;47:414‐433. 20472804 10.1177/0300985810363902

[4] Patnaik A , Hurvitz A , Johnson G . Canine gastrointestinal neoplasms. Vet Pathol. 1977;14:547‐555. 579266 10.1177/030098587701400602

[5] Zandvliet M . Canine lymphoma: a review. Vet Q. 2016;36:76‐104. 26953614 10.1080/01652176.2016.1152633

[6] Rassnick K , Moore A , Collister K , et al. Efficacy of combination chemotherapy for treatment of gastrointestinal lymphoma in dogs. J Vet Intern Med. 2009;23:317‐322. 19192147 10.1111/j.1939-1676.2008.0270.x

[7] Frank JD , Reimer SB , Kass PH , Kiupel M . Clinical outcomes of 30 cases (1997–2004) of canine gastrointestinal lymphoma. J Am Anim Hosp Assoc. 2007;43:313‐321. 17975213 10.5326/0430313

[8] Sogame N , Risbon R , Burgess KE . Intestinal lymphoma in dogs: 84 cases (1997–2012). J Am Vet Med Assoc. 2018;252:440‐447. 29393741 10.2460/javma.252.4.440

[9] Couto CG , Rutgers HC , Sherding RG , Rojko J . Gastrointestinal lymphoma in 20 dogs: a retrospective study. J Vet Intern Med. 1989;3:73‐78. 2715959 10.1111/j.1939-1676.1989.tb03082.x

[10] Ruslander D , Gebhard D , Tompkins M , et al. Immunophenotypic characterization of canine lymphoproliferative disorders. In Vivo (Athens, Greece). 1997;11:169‐172. 9179611

[11] Chun R , Garrett LD , Vail DM . Evaluation of a high‐dose chemotherapy protocol with no maintenance therapy for dogs with lymphoma. J Vet Intern Med. 2000;14:120‐124. 10772481 10.1892/0891-6640(2000)014<0120:eoahcp>2.3.co;2

[12] Beaver LM , Strottner G , Klein MK . Response rate after administration of a single dose of doxorubicin in dogs with B‐cell or T‐cell lymphoma: 41 cases (2006–2008). J Am Vet Med Assoc. 2010;237:1052‐1055. 21034344 10.2460/javma.237.9.1052

[13] Coyle K , Steinberg H . Characterization of lymphocytes in canine gastrointestinal lymphoma. Vet Pathol. 2004;41:141‐146. 15017027 10.1354/vp.41-2-141

[14] Miura T , Maruyama H , Sakai M , et al. Endoscopic findings on alimentary lymphoma in 7 dogs. J Vet Med Sci. 2004;66:577‐580. 15187374 10.1292/jvms.66.577

[15] Ruiz G , Gomez ER , Yaguiyan‐Colliard L . Exceptionally long‐term survival of a dog with gastric lymphoma and concurrent dietary intolerance. Vet Rec Case Rep. 2015;3:e000137.

[16] Borska P , Husnik R , Fictum P , Kehl A , Leva L , Faldyna M . Gastrointestinal B‐lymphoblastic lymphoma in a dog: a case report. Vet Med. 2018;63:40‐49.

[17] Van den Steen N , Berlato D , Polton G , et al. Rectal lymphoma in 11 dogs—a retrospective study. J Small Anim Pract. 2012;53:586‐591. 22882127 10.1111/j.1748-5827.2012.01258.x

[18] Desmas I , Burton JH , Post G , et al. Clinical presentation, treatment and outcome in 31 dogs with presumed primary colorectal lymphoma (2001–2013). Vet Comp Oncol. 2017;15:504‐517. 27028211 10.1111/vco.12194

[19] Kodama A , Sakai H , Kobayashi K , et al. B‐cell intestinal lymphoma with Mott cell differentiation in a 1‐year‐old miniature Dachshund. Vet Clin Pathol. 2008;37:409‐415. 19055576 10.1111/j.1939-165X.2008.00067.x

[20] Russell W . An address on a characteristic organism of cancer. Br Med J. 1890;2:1356‐1360. 10.1136/bmj.2.1563.1356 PMC2208600 20753194

[21] Mott FW . Observations on the brains of men and animals infected with various forms of trypanosomes. Proc R Soc Lond Ser B. 1905;76:235‐242.

[22] Snyman H , Fromstein J , Vince A . A rare variant of multicentric large B‐cell lymphoma with plasmacytoid and Mott cell differentiation in a dog. J Comp Pathol. 2013;148:329‐334. 23079101 10.1016/j.jcpa.2012.08.002

[23] Stacy NI , Nabity MB , Hackendahl N , et al. B‐cell lymphoma with Mott cell differentiation in two young adult dogs. Vet Clin Pathol. 2009;38:113‐120. 19171017 10.1111/j.1939-165X.2008.00101.x

[24] De Zan G , Zappulli V , Cavicchioli L , et al. Gastric B‐cell lymphoma with Mott cell differentiation in a dog. J Vet Diagn Invest. 2009;21:715‐719. 19737772 10.1177/104063870902100521

[25] Seelig DM , Perry JA , Zaks K , Avery AC , Avery PR . Monoclonal immunoglobulin protein production in two dogs with secretory B‐cell lymphoma with Mott cell differentiation. J Am Vet Med Assoc. 2011;239:1477‐1482. 22087724 10.2460/javma.239.11.1477

[26] Gupta A , Gumber S , Schnellbacher R , Bauer RW , Gaunt SD . Malignant B‐cell lymphoma with Mott cell differentiation in a ferret (Mustela putorius furo). J Vet Diagn Invest. 2010;22:469‐473. 20453231 10.1177/104063871002200326

[27] Comazzi S , Marelli S , Cozzi M , et al. Breed‐associated risks for developing canine lymphoma differ among countries: an European canine lymphoma network study. BMC Vet Res. 2018;14:1‐7. 30081964 10.1186/s12917-018-1557-2 PMC6090884

[28] Wilson‐Robles H , Budke C , Miller T , et al. Geographical differences in survival of dogs with non‐Hodgkin lymphoma treated with a CHOP based chemotherapy protocol. Vet Comp Oncol. 2017;15:1564‐1571. 28419683 10.1111/vco.12302

